# Phylogenetic group, antibiotic resistance, virulence gene, and genetic diversity of *Escherichia coli* causing bloodstream infections in Iran

**DOI:** 10.3389/fmicb.2024.1426510

**Published:** 2024-07-19

**Authors:** Saeed Hemati, Shahnaz Halimi, Fereshteh Jabalameli, Mohammad Emaneini, Reza Beigverdi

**Affiliations:** Department of Microbiology, School of Medicine, Tehran University of Medical Sciences, Tehran, Iran

**Keywords:** *Eshcerichia coli*, bloodstream infections, phylogenetic group, sequence type, virulence, antimicrobial resistance, REP-PCR

## Abstract

*Escherichia coli* is one of the most important pathogens causing bloodstream infections (BSIs) throughout the world. We sought to characterize the phylogroup classification, major human sequence types (STs), antimicrobial resistance, presence of selected antimicrobial resistance and virulence genes, and genetic diversity of *E. coli* isolated from patients with BSIs at the University Hospital in Iran. A total of 100 *E. coli* bloodstream isolates were collected between December 2020 and June 2022. This study used PCR to investigate phylogenetic groups (A, B1, B2, C, D, E, and F), four major STs (ST69, ST73, ST95, and ST131), antibiotic resistance genes (ARGs), virulence-associated genes (VAGs), and pathogenicity islands (PAIs). Antimicrobial susceptibility testing was done by disk diffusion method. Genetic diversity was analyzed by repetitive element sequence-based PCR (REP-PCR). The phylogenetic group B2 (32%) predominated, followed by phylogenetic group E (25%). ST131 (28%) was the most prevalent ST and the majority of these isolates (89.3%) were of serotype O25b. Most of *E. coli* isolates (75%) were categorized as multidrug resistant (MDR) with high rates of resistance (>55%) to ampicillin, trimethoprim-sulfamethoxazole, ciprofloxacin, cefazolin, and ceftriaxone. The most frequent ARGs were *bla*_TEM_ (66%), *sul1* (57%), and *sul2* (51%). The most prevalent VAGs and PAIs were *fimH* (type 1 fimbriae adhesin; 85%), *aer* (*iucC*) (aerobactin; 79%), *traT* (serum resistance; 77%), *iutA* (aerobactin siderophore receptor; 69%), and PAI IV_536_ (75%), respectively. The highest rate of ARGs and VAGs was observed in the ST131 isolates. REP-PCR analysis showed high diversity among the studied isolates. The high prevalence of MDR septicemic *E. coli* with different types of ARGs, VAGs and genotypes is an extremely worrisome sign of BSIs treatment and poses a major threat for hospitalized patients. Active surveillance, stringent prescribing policies, increasing the awareness of ARGs among clinicians and re-defining the infection control measures are essential to curb the dissemination of these strains.

## Introduction

*Escherichia coli* is responsible for a wide variety of diseases such as urinary tract infections (UTIs), pneumonia, bloodstream infections (BSIs), and meningitis in humans in both developed and developing countries (Mathers et al., 2015a; Sarowska et al., 2019). *E. coli* BSIs (EC-BSIs) have been associated with prolonged hospital stays, high mortality, and costs (Chen et al., 2021). Based on phylogenetic group classification, *E. coli* was classified into 7 groups (A, B1, B2, C, D, E, and F) (Zhao et al., 2021). EC-BSIs commonly belonged to group B2, and to a lesser extent, group D, while groups A and B1 were frequently associated with the commensal and less virulent strains (Cole et al., 2019). Invasive *E. coli* strains have various virulence-associated genes (VAGs) including adhesins, toxins, siderophores and capsular antigens, which allow to colonize the host and invade the bloodstream (Ciesielczuk et al., 2015). Molecular studies revealed that four clones or sequence types (STs) 69, 73, 95, and 131 were commonly responsible for most *E. coli* infections in humans (Hojabri et al., 2019b). In a UK study, it was shown that these four STs were responsible for a large proportion of UTIs and BSIs (Doumith et al., 2015). Antibiotic resistance patterns associated with these clones differ markedly: isolates belonging to STs 69, 73, and 95 are mainly susceptible to antibiotics and rarely have resistance to extended-spectrum cephalosporins, while members of ST131 are connected with high rates of cephalosporins or fluoroquinolones resistance (Doumith et al., 2015). Rapid identification of these major STs by molecular techniques can assist clinicians to select appropriate antimicrobials for treatment before conventional susceptibility test results become available (Doumith et al., 2015). Recently, EC-BSIs have been increasing in multiple countries and of greater concern is the emergence of multidrug resistant (MDR) strains, which can cause inappropriate empirical treatment (Gladstone et al., 2020). Different typing techniques have been developed for epidemiological relationships of *E. coli* (Fratamico et al., 2016). Among polymerase chain reaction (PCR)-based approaches, repetitive extra-genic palindromic elements (REP)-PCR is a rapid, simple, reliable and cost-effective method with high discriminatory power compared to other genotyping techniques (Sabat et al., 2013). Information about the molecular epidemiology of EC-BSIs is limited in Iran. Therefore, the present study aimed to evaluate phylogroup classification, four major human STs, antimicrobial resistance, presence of selected antimicrobial resistance and virulence genes, and also genetic diversity in a teaching hospital of Tehran, Iran.

## Materials and methods

### Study design and bacterial isolates

This cross-sectional study was performed in a 640-beds university-affiliated hospital (Shariati hospital) in Iran between December 2020 and June 2022, using a total of 100 non-duplicate (one isolate per patient) *E. coli* were isolated from blood samples of patients with clinical symptoms of BSIs. *E. coli* was identified using colony morphology and biochemical profile by the standard laboratory guidelines (Pathak et al., 2013). The isolates were stored at −70°C in Trypticase Soy Broth (TSB) containing 30% glycerol until further characterization.

### Phylogenetic classification and sequence-typing PCR

Genomic bacterial DNA was extracted from freshly cultured bacteria by boiling method (Iranpour et al., 2015). Major *E. coli* phylogenetic groups (A, B1, B2, C, D, E, or F) were determined using multiplex PCR (Clermont et al., 2013). The four main *E. coli* STs (ST69, ST73, ST95, and ST131) were evaluated by PCR as previously described (Doumith et al., 2015). Furthermore, molecular serotyping of ST131 isolates was performed by PCR method to detect two common serogroups, including O16 and O25b (Park et al., 2018).

### Antimicrobial susceptibility assay

Antibiotic susceptibility testing was performed using a disk-diffusion method on Mueller-Hinton agar according to Clinical Laboratory Standard Institute (CLSI) 2022 guidelines. The following antimicrobial agents were tested in this study: ampicillin, trimethoprim-sulfamethoxazole, ciprofloxacin, piperacillin-tazobactam, cefazolin, ceftriaxone, cefepime, ceftazidime, gentamicin, ampicillin-sulbactam, imipenem, meropenem, and amikacin. MDR refers an isolate resistant to at least one potentially effective antimicrobial drug in three or more classes (such as penicillins/cephalosporins, fluoroquinolones, carbapenems, and aminoglycosides) (Tam et al., 2010; Zeighami et al., 2019). Extensively drug-resistant (XDR) was defined as those that were resistant to all except one or two antimicrobial classes (Falagas et al., 2010). The resistance score was calculated by the number of resistant antimicrobial agents for each isolate. A score of 1 shows resistant; 0.5, intermediary; and 0, sensitive (Duan et al., 2020).

### Detection of antibiotic resistance genes

Molecular determination of ARGs was carried out by PCR based on amplification of genes with specific primers previously described. Isolates were screened for extended-spectrum beta-lactamases (ESBLs) genes (*bla*_CTX-M_, *bla*_TEM_, and *bla*_SHV_) (Neyestanaki et al., 2014), plasmid-mediated quinolone resistance genes (*qnrA* and *qnrB*) (Wu et al., 2007), plasmid-mediated trimethoprim resistance gene (*dfrA*) (Boroumand et al., 2021), plasmid-mediated sulfonamide resistance genes (*sul1*, *sul2*, and *sul3*) (Jiang et al., 2019), carbapenemase genes (*bla*_OXA_, *bla*_NDM_, *bla*_IMP_, *bla*_VIM_, *bla*_SPM_ and *bla*_KPC_) (Neyestanaki et al., 2014; Bian et al., 2019), and plasmid-mediated colistin resistance gene (*mcr-1*) (Peng et al., 2019). Furthermore, *bla*_CTX-M_ and *bla*_OXA_ positive isolates were screened for *bla*_OXA-48_, *bla*_OXA-181_ (Sheikh et al., 2021), *bla*_CTX-M-14_ and *bla*_CTX-M-15_ (Tawfik et al., 2011). The resistance-gene score was the number of unique ARGs detected for each isolate.

### Identification of VAGs and pathogenicity islands

PCR was performed to screen 13 different VAGs, including adhesins (*fimH*, *papC*, *papG* II, *papG* III, and *sfa/foc*) (Yun et al., 2014), toxins (*cnf-1*, *cvaC*, and *hlyC*) (Dezfulian et al., 2003), capsules (*rfc* and *kpsMTII*) (Chapman et al., 2006), siderophores (*aer* [*iucC*] and *iutA*) (Guiral et al., 2015), serum resistance factor (*traT*) (Rasoulinasab et al., 2021), and 8 PAIs (PAI I_536_, PAI II_536_, PAI III_536_, PAI IV_536_, PAI ICFT_073_, PAI IICFT_073_, PAI I_J96_, and PAI II_J96_) (Rasoulinasab et al., 2021). The virulence-gene score was calculated by the number of the virulence genes detected for each isolate (Merino et al., 2020).

### Repetitive extragenic palindromic element PCR genotyping

REP-PCR method was performed using a T100^™^ Thermal Cycler (BioRad, Germany) instrument with primers rep-F (5′-ICGICTTAT CIGGCCTAC-3′) and rep-R (5′-IIIICGICGICATCIGGC-3′). Amplification and electrophoresis were carried out as described previously (Erfanimanesh et al., 2022). In the lack of appropriate software, the band patterns generated by REP-PCR were assessed visually. The binary matrix was built based on the absence or presence of each band (coded 0 or 1, respectively) (Pusparini et al., 2018). Phylogenetic tree based on binary matrix was constructed by the free online service at http://insilico.ehu.eus/dice_upgma/. Isolates were classified together into the same cluster when their similarity coefficient was ≥80%.

### Data analysis

Statistical analyses were performed using SPSS software (version 16.0). Comparison between the variables was performed using Pearson’s Chi-square test or Fisher’s exact test. The differences were considered statistically significant if *p*-value ≤ 0.05. Correlation analyses (Spearman *R* correlation coefficient) were performed to evaluate the correlation between the variables, demonstrated with correlation heatmaps. Cut off value was considered Spearman *R* ≥ 0.4 (Rezatofighi et al., 2021). The preparation of heatmaps was done on R software (version 4.3.2).

## Results

### Phylogenetic group and ST distribution

Phylogenetic analysis showed that 32% of our isolates belonged to phylogenetic group B2, 25% to group E, 13% to group C, 11% to group B1, 9% to group A, 7% to group F, and 3% to group D. Analysis of major *E. coli* STs indicated that the most prevalent ST was ST131 (28%, *n* = 28/100), followed by ST69 (17%, *n* = 17/100) and ST95 (2%, *n* = 2/100). ST73 was not found. Fifty-three (53%, *n* = 53/100) isolates were unknown STs. Distribution of STs in different phylogenetic groups was as follow: group B2 (ST131: 78.6%, *n* = 22; ST95: 100%, *n* = 2; unknown STs: 15.1%, *n* = 8); group E (ST69: 64.7%, *n* = 11; unknown STs: 26.4%, *n* = 14); group C (ST131: 10.8%, *n* = 3; ST69: 5.9%, *n* = 1; unknown STs: 17%, *n* = 9); group B1 (ST131: 7.1%, *n* = 2; unknown STs: 17%, *n* = 9); group A (ST69: 11.8%, *n* = 2; unknown STs: 13.2%, *n* = 7); group F (ST69: 11.8%, n = 2; unknown STs: 9.4%, *n* = 5); group D (ST131: 3.5%, *n* = 1; ST69: 5.9%, *n* = 1; unknown STs: 1.9%, *n* = 1). The majority of ST131 and all ST95 isolates belonged to phylogenetic group B2, while most ST69 isolates belonged to phylogenetic group E. PCR O-typing of 28 ST131 isolates revealed that the vast majority of the isolates (25/28, 89.3%) harbored O25b and 10.7% (3/28) had O16.

### ST distribution in relation to patient’s age and gender

The median age of the 100 patients was 67 years old (range 0–86 years). Thirty-eight (38%) patients were female (mean age 61 years, range 16–87 years), and 62 (62%) patients were male (mean age 54 years, range 0–86 years). Sixty-nine (69%) were aged over 50 years and one (1%) of patients was aged less than 1 year. Most (60.7%, *n* = 17) ST131 isolates were predominant in females, followed by unknown STs (30.2%, *n* = 16) and ST69 (29.4%, *n* = 5) (*p* = 0.01); whereas, ST95 (100%, *n* = 2) isolates predominated in males, followed by ST69 (70.6%, *n* = 12), unknown STs (69.8%, *n* = 37), and ST131 (39.3%, *n* = 11) (*p* = 0.01). Elderly patients over 50 years were more frequent; however, a significant association was not observed with the patterns of ST distribution (*p* = 0.1).

### Antibiotic susceptibility in relation to ST background

Antimicrobial susceptibility testing results revealed that more than 55% of our isolates were resistant to ampicillin (89%), trimethoprim-sulfamethoxazole (75%), ciprofloxacin (71%), cefazolin (64%), and ceftriaxone (58%). In contrast, the low resistance rates were observed for carbapenems (4%) and amikacin (2%). The frequencies of MDR and XDR isolates were 75 and 5%, respectively (Table 1). Concerning the antibiotic susceptibility of ST131 and non-ST131 isolates, ST131 had higher resistance rates than non-ST131 isolates and these differences were significant for cephalosporins, gentamicin, and amikacin (*p* < 0.05). Moreover, most ST131 isolates (92.9%) were MDR.

**Table 1:** Antimicrobial resistance based on major sequence types (STs) among *Escherichia coli* isolates.

Antibiotic		No. (%) isolates by ST				
	ST69 *n* = 17	ST95 *n* = 2	ST131 *n* = 28	Unknown ST *n* = 53	Total *n* = 100	*p* value
AM	16 (94.1%)	1 (50%)	28 (100%)	44 (83.0%)	89 (89%)	
TS	13 (76.5%)	0	26 (92.5%)	36 (67.9%)	75 (75%)	
CIP	9 (52.9%)	0	23 (82.1%)	39 (73.6%)	71 (71%)	
PTZ	4 (23.5%)	0	8 (28.6%)	12 (22.6%)	24 (24%)	
CZ	11 (64.7%)	0	26 (92.5%)	27 (50.9%)	64 (64%)	0.003
CRO	8 (47.1%)	0	26 (92.5%)	24 (45.3%)	58 (58%)	0.001
CPM	6 (35.3%)	0	24 (85.7%)	18 (34%)	48 (48%)	0.001
CAZ	7 (41.2%)	0	21 (75%)	14 (26.4%)	42 (42%)	0.001
GM	1 (5.9%)	0	16 (57.1%)	11 (20.8%)	28 (28%)	0.001
FAM	3 (17.6%)	0	4 (14.3%)	11 (20.8%)	18 (18%)	
IMP	1 (5.9%)	0	1 (3.6%)	2 (3.8%)	4 (4%)	
MEM	1 (5.9%)	0	1 (3.6%)	2 (3.8%)	4 (4%)	
AK	0	0	2 (7.1%)	0	2 (2%)	0.03
MDR	12 (70.6%)	0	26 (92.9%)	37 (69.8%)	75 (75%)	0.01
XDR	1 (5.9%)	0	1 (3.6%)	3 (5.7%)	5 (5%)	0.02
**Resistance scores**		**Score median (mean, range)**				
	4 (4.7, 11)	0.5 (0.5, 1)	8 (7.35, 11)	4 (4.5, 12)		0.004

AM, ampicillin; TS, trimethoprim-sulfamethoxazole; CIP, ciprofloxacin; PTZ, piperacillin-tazobactam; CZ, cefazolin; CRO, ceftriaxone; CPM, cefepime; CAZ, ceftazidime; GM, gentamicin; FAM, ampicillin-sulbactam; IMP, imipenem; MEM, meropenem; AK, amikacin.

### Detection of ARGs and relation to ST background

The overall prevalence of individual ARGs ranged from 0% (*bla*_KPC_, *bla*_IMP_, *bla*_SPM_, *qnrA*, and *mcr-1*) to 66% (*bla*_TEM_) (Table 2). More than half of the isolates harbored *bla*_TEM_, *sul1*, and *sul2* genes. *bla*_CTX-M_, *dfrA*, *sul1*, *bla*_OXA_, and *bla*_VIM_ genes were more frequent in ST131 isolates, while *bla*_TEM_, *qnrB* and *bla*_NDM_ genes were more common in ST69 isolates. Additionally, resistance-gene scores of the ST131 isolates were slightly higher than other non-ST131 isolates.

**Table 2:** Distribution of resistance genes among major sequence types (STs) of *Escherichia coli* isolates.

Resistance genes		No. (%) isolates by ST				
	ST69 *n* = 17	ST95 *n* = 2	ST131 *n* = 28	Unknown STs *n* = 53	Total *n* = 100	*p* value
*bla*_TEM_	12 (70.6%)	1 (50%)	16 (57.1%)	37 (69.8%)	66 (66%)	
*bla*_SHV_	0	1 (50%)	1 (3.6%)	2 (3.8%)	4 (4%)	
*bla*_CTX-M_	0	1 (50%)	16 (57.1%)	7 (13.2%)	24 (24%)	0.001
*bla*_CTX-M-14_	0	0	3 (10.7%)	1 (1.9%)	4 (4%)	0.001
*bla*_CTX-M-15_	0	0	13 (46.4%)	6 (11.3%)	19 (19%)	0.001
*qnrB*	2 (11.8%)	0	1 (3.6%)	4 (7.5%)	7 (7%)	
*dfrA*	1 (5.9%)	0	5 (17.9%)	6 (11.3%)	12 (12%)	
*sul1*	12 (70.6%)	1 (50%)	21 (75%)	23 (43.4%)	57 (57%)	
*sul2*	7 (41.2%)	1 (50%)	14 (50%)	29 (54.7%)	51 (51%)	
*sul3*	0	1 (50%)	1 (3.6%)	12 (22.6%)	14 (14%)	
*bla*_NDM_	1 (5.9%)	0	1 (3.6%)	2 (3.8%)	4 (4%)	
*bla*_OXA_	4 (23.5%)	0	14 (50%)	6 (11.3%)	24 (24%)	0.001
*bla*_OXA-48_	0	0	1 (3.6%)	1 (1.9%)	2 (2%)	0.001
*bla*_OXA-181_	3 (17.6%)	0	4 (14.3%)	1 (1.9%)	8 (8%)	0.001
*bla*_VIM_	1 (5.9%)	0	7 (25%)	3 (5.7%)	11 (11%)	0.01
**Resistance-gene scores**		**Score median (mean, range)**				
	3 (2.5, 5)	4 (4, 2)	4 (3.9, 5)	3 (2.6, 6)		0.009

### Association between the ARGs and antimicrobial resistance phenotype

**Figure 1:**
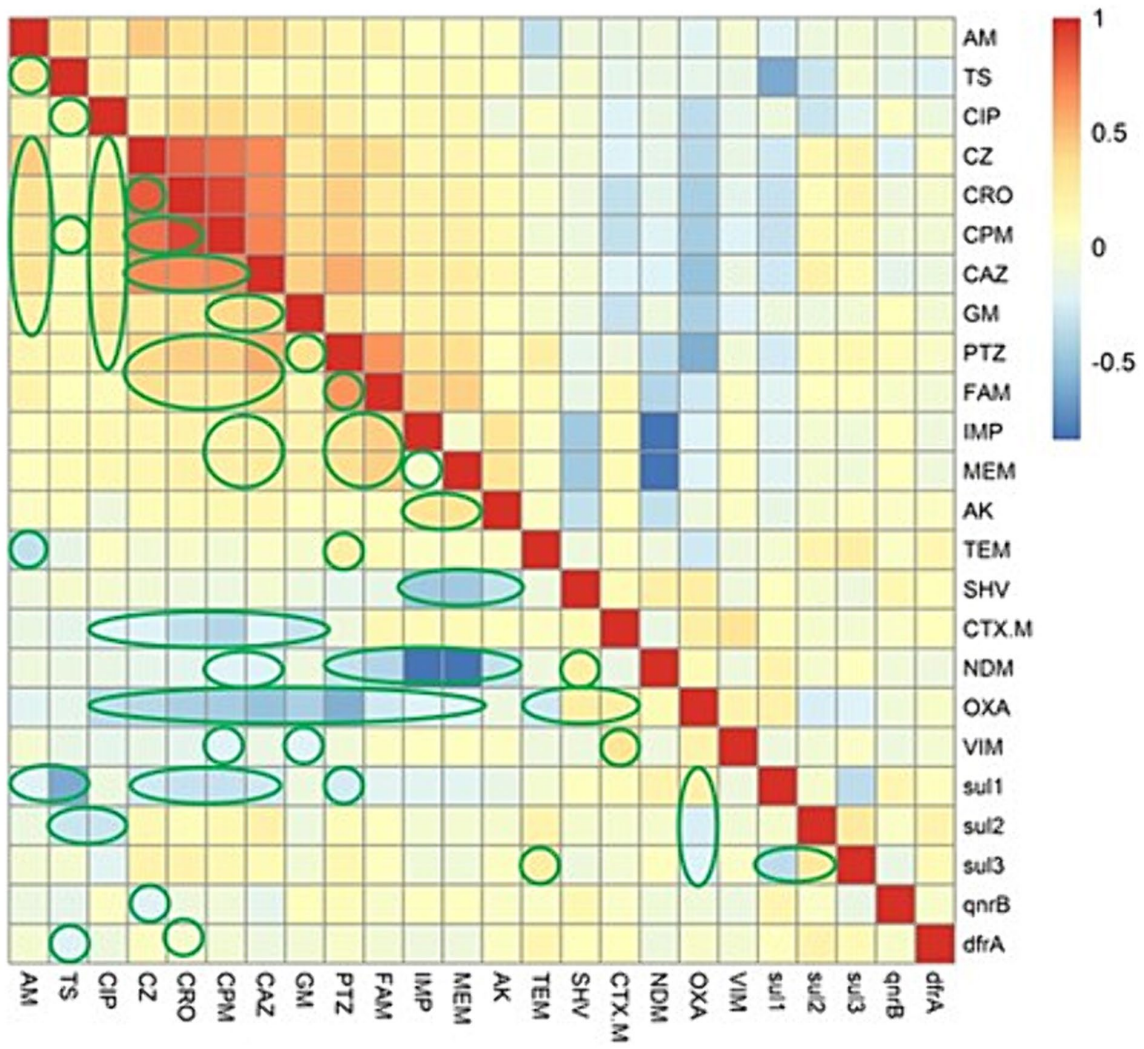
The heatmap demonstrates the spearman correlation among various antimicrobial agents and the antimicrobial resistance genes in *Escherichia coli* causing bloodstream infections. The statistically significant associations (*p* < 0.05) are indicated by closed green lines in the heatmap cells. The red color represents the positive correlation, the darker red denotes a stronger positive correlation coefficient, the blue color represents negative correlation, and the darker blue denotes a stronger negative correlation coefficient. AM, ampicillin; TS, trimethoprim-sulfamethoxazole; CIP, ciprofloxacin; PTZ, piperacillin-tazobactam; CZ, cefazolin; CRO, ceftriaxone; CPM, cefepime; CAZ, ceftazidime; GM, gentamicin; FAM, ampicillin-sulbactam; IMP, imipenem; MEM, meropenem; AK, amikacin.

We evaluated association between the ARGs and AMR phenotype by Spearman’s rank correlation coefficient. As demonstrated in the correlation matrix in Figure 1, no strong positive association was observed between them in the present study. While, it was found that there is a weak positive association between the *bla*_TEM_ gene and resistance to piperacillin-tazobactam. We also found a weak positive association between the resistance genes, including *sul3* with *sul1* and *sul2* with *bla*_OXA_. Furthermore, analysis of the co-resistance phenomenon showed a strong positive association between the following antibiotics: (cefazolin with ampicillin, ceftriaxone, cefepime, ceftazidime, and piperacillin-tazobactam); (ceftriaxone with cefepime, ceftazidime, and piperacillin-tazobactam); (cefepime with ceftazidime, gentamicin, and piperacillin-tazobactam); (ceftazidime with gentamicin, ampicillin-sulbactam, and piperacillin-tazobactam); (piperacillin-tazobactam with ampicillin-sulbactam); (ampicillin-sulbactam with imipenem and meropenem).

### Detection of virulence genes and relation to ST background

The overall prevalence of VAGs ranged from 0% (*papG* III) to 85% (*fimH*). Five virulence genes (*fimH*, *aer* [*iucC*], *traT*, *iutA,* and *kpsMT* II) were found in ≥56% of the isolates. In contrast, four VAGs (*hlyC*, *cnf-1*, *cvaC*, and *sfa*/*foc*) were found in less than 16% of the isolates (Table 3). In this study, the most predominant PAI marker was PAI IV_536_ (75%), followed by PAI IICFT_073_ (30%), and PAI ICFT_073_ (28%), whereas three PAIs (PAI II_J96,_ PAI II_536_ and PAI I_536_) were found in less than 13% of the isolates. Two PAIs (PAI I_J96_ and PAI III_536_) were not detected in any isolate. A number of VAGs (*papC*, *papG* II, *cnf-1*, *hlyC*, *kpsMTII*, *aer* [*iucC*], *iutA*, and *traT*) and PAIs (other than PAI IV_536_) were observed significantly more frequently in ST131 isolates compared with non-ST131 isolates. Statistical analysis showed that VAGs were more prevalent among phylogroup B2 isolates (mean: 9.6, median: 8.0), followed by group D (mean: 7.3, median: 7.0), group E (mean: 6.2, median: 6.0), group C (mean: 4.9, median: 5.0), group F (mean: 4.1, median: 5.0), group B1 (mean: 4.4, median: 4.0), and group A (mean: 3.2, median: 3.0) (*p* < 0.001).

**Table 3:** Distribution of virulence genes among major sequence types (STs) of *Escherichia coli* isolates.

Virulence genes	Description	No. (%) isolates by ST					
		ST69 (*n* = 17)	ST95 (*n* = 2)	ST131 (*n* = 28)	Unknown STs (*n* = 53)	Total (%) *n* = 100	*p* value
*fimH*	Type 1 fimbriae adhesin	16 (94.1%)	2 (100%)	26 (92.9%)	41 (77.4%)	85 (85%)	
*traT*	Outer membrane lipoprotein	14 (82.4%)	2 (100%)	24 (85.7%)	37 (69.8%)	77 (77%)	
*iutA*	Aerobactin siderophore receptor	10 (58.8%)	1 (50%)	25 (89.3%)	33 (62.3%)	69 (69%)	0.01
*papC*	P fimbriae C	7 (41.2%)	2 (100%)	13 (46.4%)	14 (26.4%)	36 (36%)	
*papG* II	P fimbriae (G II)	7 (41.2%)	2 (100%)	18 (64.3%)	14 (26.4%)	41 (41%)	0.003
*sfa/foc*	s-fimbrial and F1C fimbriae adhesin	1 (5.9%)	0	2 (7.1%)	4 (7.5%)	7 (7%)	
*cnf-1*	Cytotoxic necrotizing factor 1	0	0	10 (35.7%)	0	10 (10%)	0.001
*cvaC*	Colicin C	4 (23.5%)	0	0	5 (9.4%)	9 (9%)	0.01
*hlyC*	Hemolysin C	1 (5.9%)	1 (50%)	10 (35.7%)	3 (5.7%)	15 (15%)	0.01
*rfc*	O4 lipopolysaccharide synthesis	3 (17.6%)	1 (50%)	5 (17.9%)	13 (24.5%)	22 (22%)	
*kpsMT* II	Group capsular II	10 (58.8%)	2 (100%)	22 (78.6%)	22 (41.5%)	56 (56%)	0.007
*aer* (*iucC*)	Aerobactin	15 (88.2%)	2 (100%)	26 (92.2%)	36 (67.9%)	79 (79%)	0.07
PAI I_536_	Pathogenicity island	0	0	7 (25%)	0	7 (7%)	0.001
PAI II_536_	Pathogenicity island	0	0	10 (35.7%)	0	10 (10%)	0.001
PAI IV_536_	Pathogenicity island	17 (100%)	2 (100%)	25 (89.3%)	31 (58.5%)	75 (75%)	
PAI ICFT_073_	Pathogenicity island	3 (17.6%)	2 (100%)	16 (57.1%)	7 (13.2%)	28 (28%)	0.001
PAI IICFT_073_	Pathogenicity island	0	0	25 (89.3%)	5 (9.4%)	30 (31%)	0.001
PAI II_J96_	Pathogenicity island	0	0	9 (32.1%)	3 (5.7%)	12 (12%)	0.001
**Virulence gene scores**		**Score median (mean, range)**					
		7 (6.3, 6)	9.5 (9.5, 3)	8 (9.7, 12)	5 (5, 11)		0.01

### Association between the VAGs and PAI markers

**Figure 2:**
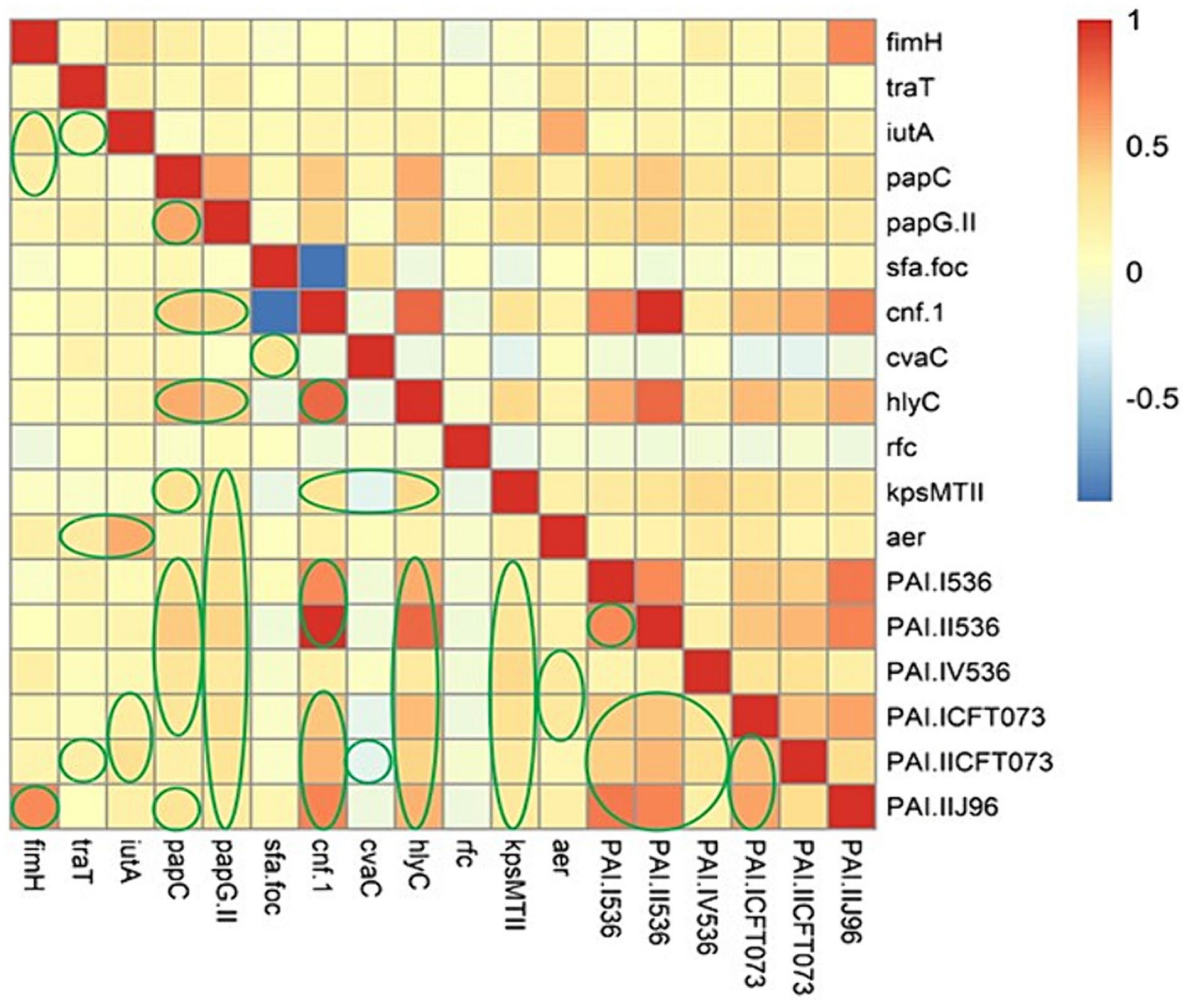
The heatmap illustrates the spearman correlation between different virulence markers in *Escherichia coli* causing bloodstream infections. The statistically significant associations (*p* < 0.05) are indicated by closed green lines in the heatmap cells. The red color represents the positive correlation, the darker red denotes a stronger positive correlation coefficient, the blue color represents negative correlation, and the darker blue denotes a stronger negative correlation coefficient.

We performed Spearman’s rank correlation coefficient analysis to evaluate the association between the virulence determinants in EC-BSIs. On the basis of Figure 2, Spearman correlation analysis displayed strong positive associations among the following genes: (*fimH* with PAI II_J96_); (*iutA* with *aer* [*iucC*]); (*papC* with *papG* II, *hlyC*, *cnf*-*1*, and PAI II_536_); (*papG* II with *cnf*-*1*, *papC* and *hlyC*); (*cnf*-*1* with *hlyC*, PAI I_536_, PAI II_536_, PAI ICFT_073_, PAI IICFT_073_, and PAI II_J96_); (*hlyC* with PAI I_536_, PAI II_536_, PAI ICFT_073_, and PAI II_J96_); (PAI I_536_ with PAI II_536_, PAI ICFT_073_, PAI IICFT_073_, and PAI II_J96_); (PAI II_536_ with PAI ICFT_073_, PAI IICFT_073_, and PAI II_J96_); (PAI ICFT_073_ with PAI IICFT_073_, and PAI II_J96_).

### Association between the virulence markers and antibiotic resistance

**Figure 3:**
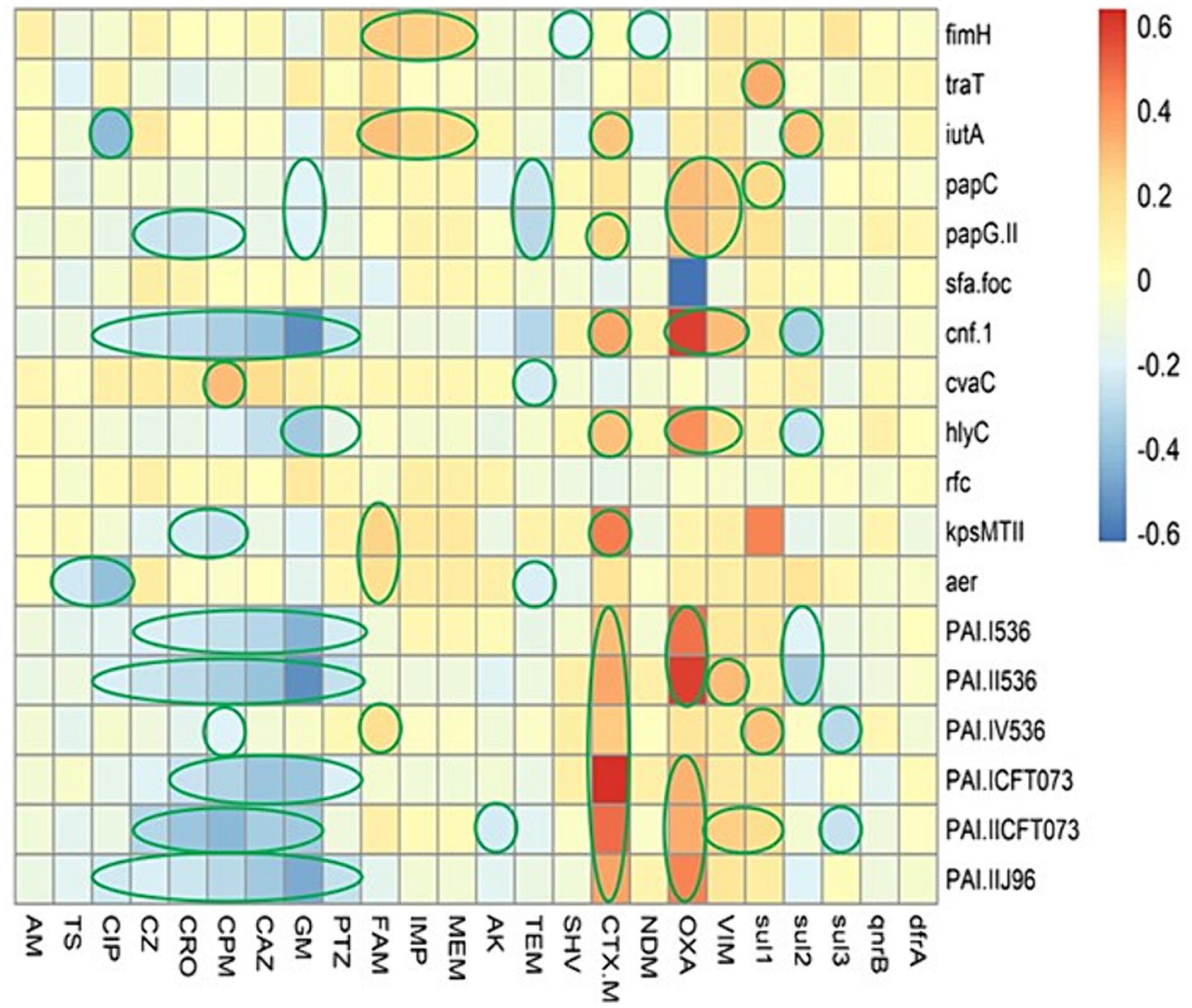
The heatmap demonstrates the spearman correlation between various virulence markers and antimicrobial resistance phenotype in *Escherichia coli* causing bloodstream infections. The statistically significant associations (*p* < 0.05) are indicated by closed green lines in the heatmap cells. The red color represents the positive correlation, the darker red denotes a stronger positive correlation coefficient, the blue color represents negative correlation, and the darker blue denotes a stronger negative correlation coefficient. AM, ampicillin; TS, trimethoprim-sulfamethoxazole; CIP, ciprofloxacin; PTZ, piperacillin-tazobactam; CZ, cefazolin; CRO, ceftriaxone; CPM, cefepime; CAZ, ceftazidime; GM, gentamicin; FAM, ampicillin-sulbactam; IMP, imipenem; MEM, meropenem; AK, amikacin.

In this regard, we were not observed any strong positive association between the AMR phenotype and virulence factors (Figure 3); however, a weak positive association was observed as follow: (ampicillin-sulbactam with *fimH*, *iutA*, *kpsMTII*, *iucC*, and PAI IV_536_); (imipenem with *fimH*, and *iutA*); (meropenem with *fimH*, and *iutA*). Strong positive associations were detected between the presence of some ARGs and VAGs, including: (*bla*_CTX-M_ with *kpsMTII*, PAI ICFT_073_, and PAI IICFT_073_); (*bla*_OXA_ with *cnf-1*, *hlyC*, PAI I_536_, PAI II_536_, and PAI II_J96_).

### Genetic relatedness analysis

**Figure 4:**
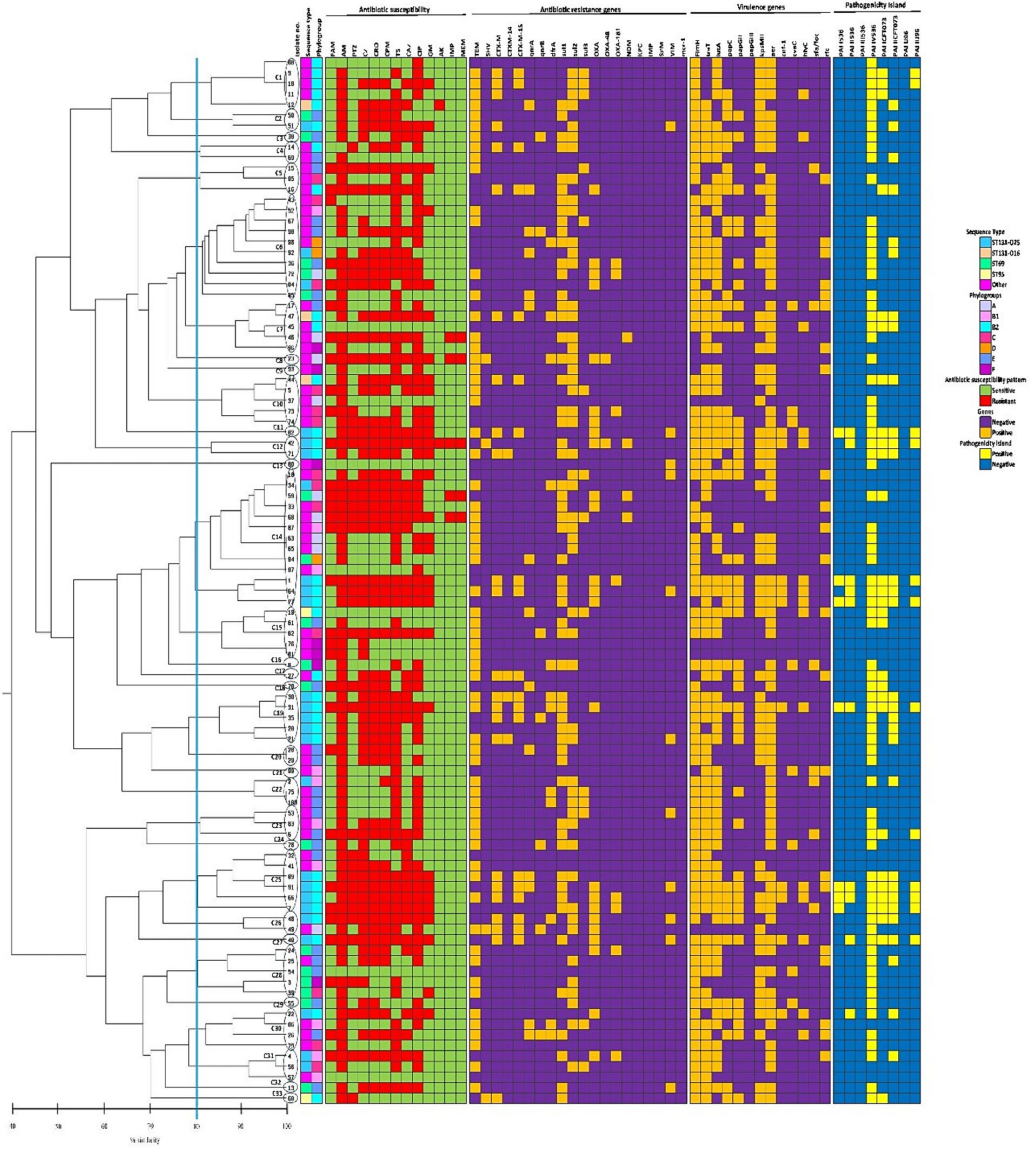
The phenotypic and genotypic characteristics of 99 *Escherichia coli* bloodstream infection isolates included in the present study. The left side of the figure is the REP-PCR dendrogram and the right side is the heat map of isolates based on their antimicrobial resistance patterns, the distribution of antibiotic resistance genes, virulence-associated genes, and pathogenicity islands which are color-coded feature categories. The vertical line displays the 80% similarity cut-off value. Based on a similarity index ≥80%, 33 clusters were found. Numbers at the terminal branches are strain name. Phylogenetic tree generated with DNA banding patterns based on REP primers using unweighted-pair group method with arithmetic mean cluster analysis. AM, ampicillin; TS, trimethoprim-sulfamethoxazole; CIP, ciprofloxacin; PTZ, piperacillin-tazobactam; CZ, cefazolin; CRO, ceftriaxone; CPM, cefepime; CAZ, ceftazidime; GM, gentamicin; FAM, ampicillin-sulbactam; IMP, imipenem; MEM, meropenem; AK, amikacin.

REP-PCR technique produced varying amplification products from about 100 to about 3,000 bp with different bands (between 1 and 13 bands) for each isolate. According to Figure 4, REP-PCR analysis displayed 91 distinct genetic patterns which clustered the isolates into 33 REP types (named as C1-C33). No band was observed in one isolate. C14 was the most abundant cluster, comprising 13.14% (13/99) of the isolates, followed by C6 (10.1%, 10/99). Other clusters included <10 isolates. Comparison of isolates in the same cluster showed that most isolates were different in relation to the gene distributions and antimicrobial resistance profile. Furthermore, REP-PCR showed that the isolates belonging to the same STs, exhibited a wide range of genetic diversity.

## Discussion

In the current study, we investigated 100 clinical *E. coli* isolates from BSIs for phylogenetic group distributions, four major STs, virulence/resistance genes as well as the genetic relatedness. In our study, the phylogenetic classification indicated that phylogenetic groups B2 and E were the predominant groups. In 2023, Li et al. analyzed 60 *E. coli* bloodstream isolates in China and found that phylogroups B2 and D were the most prevalent groups (Li et al., 2023). In 2019, Daga et al. studied 48 *E. coli* bloodstream isolates in Brazil and observed that phylogroups B2 and B1 were the most common one (Daga et al., 2019). Previous studies have shown that phylogroup A was the most predominant among uropathogenic *E. coli* (UPEC) isolates in Russia and Egypt (Khairy et al., 2019). In this study, PCR assay for detection of four major *E. coli* STs revealed that ST131 (28%) was the predominant clone. Based on recent studies, the prevalence of ST131 varies from 12.5% to nearly 30% among human clinical isolates (Banerjee and Johnson, 2014). In a previous study in a single hospital in Semnan (Iran), Hojabri et al. reported that 29.8% of *E. coli* strains isolated from urine belonged to ST131 (Hojabri et al., 2017). A previous study conducted in China during 2020 to 2021 revealed that ST131 (26.7%) was the most common clone in blood samples (Li et al., 2023). In Korean study, Park et al. showed that ST131 (11.9%) predominated among *E. coli* bloodstream isolates (Park et al., 2018). Similar to previous studies, we observed that O25b was the predominant serotype among the studied ST131 isolates (Hojabri et al., 2017; Namaei et al., 2017; Zhong et al., 2019). In contrast, Zhong et al. reported that O16 was the most prevalent serotype (75%) among ST131 isolates (Zhong et al., 2015). As mentioned earlier, the distribution of phylogenetic groups, STs and serotypes varies between different countries and these discrepancies may be due to the geographical variation, origin of isolates, differences in the sample size and host populations (Zhang et al., 2002). Regarding antibiotic resistance, high rates of resistance were observed against ampicillin, trimethoprim-sulfamethoxazole, ciprofloxacin, cefazolin, and ceftriaxone. Furthermore, resistance patterns of tested isolates have shown that 75 and 5% were MDR and XDR, respectively. Similar trends have been reported in Iran; where, Hojabri et al. have observed that 90.8% of *E. coli* isolates collected from different clinical samples were MDR (Hojabri et al., 2020). On the other hand, Ny et al. have indicated that the MDR rate varies from 1.7% (Latvia) to 26.9% (Russia) among six European countries (Ny et al., 2019). A possible explanation for this variation is that inappropriate and excessive drug usage is still common in Iran, whereas in developed countries policies have been implemented to limit the use of antibiotics. In the current study, amikacin and carbapenems were the most effective antibiotic against *E. coli* than other tested antibiotics, which is in agreement with previous studies (Mazumder et al., 2020; Li et al., 2023). We also statistically compared antibiotic resistance characteristics of isolates between STs. It should be noted that antibiotic resistance of ST131 isolates was significantly higher than ST95 and ST73 isolates. This is not surprising, because *E. coli* ST131 is a pandemic MDR clone and is associated with a large proportion of antibiotic-resistant *E. coli* infections in various clinical settings (Riley, 2014; Mathers et al., 2015b). In this study, we detected different resistance genes, and the most were *bla*_TEM_ (66%), *sul1* (57%), *sul2* (51%), *bla*_CTX-M_ (24%), and *bla*_OXA_ (24%). The prevalence rate of ARGs varies widely between countries. In Palestine, Tayh et al. reported that *sul* (28.6%), *bla*_TEM_ (17%) and *bla*_OXA_ (2.4%) were the most prevalent ARGs among *E. coli* strains isolated from urine samples (Tayh et al., 2023). In a Chinese study, 18.3, 13.3 and 13.3% of the *E. coli* isolates harbored *bla*_CTX-M-15,_ *bla*_TEM_ and *bla*_OXA-1_ genes, respectively (Li et al., 2023). In Bangladesh, Mazumder et al. observed that *bla*_CTX-M-15_ (52%), *bla*_TEM_ (20) and *bla*_NDM-1_ (5%) were the most prevalent ESBL and carbapenemase genes among *E. coli* isolates (Mazumder et al., 2020). The observed variation may be attributed to the different patterns of use of antibiotics, dissemination of specific clones harboring multiple ARGs and the number of isolates studied. In current study, we found that the highest resistance score belonged to the ST131 isolates and these isolates carried multiple ARGs which could potentially complicate the medical treatment of BSIs. Notably, we observed a common phenomenon that genotypic and phenotypic resistances were not entirely consistent. In this study, of the eleven isolates harboring *bla*_VIM_ carbapenemase, only four isolates were resistant to imipenem and meropenem, while the remaining seven isolate were susceptible. This might be due to the mutations, unusual expression of ARGs or the expression of ARGs has not reached a level that can lead to drug resistance (Yu et al., 2020; Zhang et al., 2021). Generally, the antibiotic resistance phenotype relies on the carriage and expression of ARGs. The associations between AMR phenotypes and ARGs varied in this study. Mostly, negative associations were more common between different AMR and ARGs. But, we found some weak positive associations among AMR and ARGs. Positive correlations between AMR and ARGs might be attributed to the selective pressure exerted by antimicrobial agents on bacteria, leading to increased expression of ARGs or the simultaneous colocation of ARGs on single mobile genetic elements (MGEs), such as integrons, transposons, or plasmids (Birkegård et al., 2017). Nonetheless, the correlation reported in this study is based only on statistical data, and more investigations are needed to clarify the underlying mechanisms of this correlation. In contrast to the findings in our study, Rahman et al. reported that the number of ARGs is strongly associated with the phenotypic AMR in UPEC isolates (Rahman et al., 2022). Rosengren et al. reported that the agreement between AMR and ARGs varied from 33 to 85% (Rosengren et al., 2009). We found various VAGs and PAIs, of which *fimH* (85%), *iucC* (79%), *traT* (77%), PAI IV536 (75%), *iutA* (69%), and *kpsMTII* (56%) were the most prevalent. It seems that the high prevalence of these VAGs appears to be good indicators for EC-BSIs. While *papG III*, *hlyC*, *cnf-1*, *cvaC*, and *sfa/foc* genes, which were observed at low frequencies in this study, are considered to be key factors for UTIs caused by UPEC isolates (Bingen-Bidois et al., 2002; Doye et al., 2002; Whelan et al., 2023). Our findings are in agreement with the results reported by Daga et al., showing the *fimH* (95.8%), *traT* (77.1%), PAI IV_536_ (77.1%), *iutA* (64.3%), *kpsMTII* (45.8%) and to be considerably more prevalent in the septicemic *E. coli* strains (Daga et al., 2019). The current study, along with other studies from different part of the world (Cooke et al., 2010; Blanco et al., 2013; Johnson et al., 2014), shows that these virulence factors are essential for the development of *E. coli* extraintestinal infections and could be considered as vaccine candidates. Our results also revealed that the ST131 bloodstream isolates exhibited a higher virulence score than non-ST131 isolates, indicating higher virulence power and therefore the possible reason for their high prevalence among our isolates. In this study we observed that phylogroups B2 and D had high level of VFs score. On the other hand, isolates belonged to phylogroups A and B1 exhibited low level of VFs score. In agreement with this finding, other studies in the China (Li et al., 2023), Ethiopia (Dadi et al., 2020), and Pakistan (Ali et al., 2019) have shown that *E. coli* strains belong to phylogroups B2 and D possessed more VFs than the other phylogroups. However, in a study from Egypt, Khairy et al. exhibited that VFs were more common in phylogroup A (Khairy et al., 2019). This discrepancy may be related to the geographical differences and type of infections. Association between VAGs and PAIs were also analyzed. The strongest positive associations were observed between some VAGs and PAIs, suggesting the genetic linkages between them. Concerning the relationship between VAGs and AMR, two hypotheses have been postulated (Erjavec et al., 2007; Rezatofighi et al., 2021). First, resistant strains can be more virulent because mobile genetic elements (MGEs) including transposons, integrons, and plasmids may contain VAGs and AMR genes simultaneously. On the other hand, some investigators claim that AMR strains are not more virulent than susceptible ones because the evolution of AMR and virulence may not have occurred simultaneously (Erjavec et al., 2007; Rezatofighi et al., 2021). In the current study, no relationship was observed between VAGs and resistance phenotype. Only weak positive associations were found between some VAGs and resistance phenotype. The relationship between VAGs and resistance phenotypes is a complex phenomenon and influenced by numerous factors, such as geographical locations, specific hosts, and type of antimicrobial drugs used, methods used to identify VAGs and evaluate antibiotic resistance, genetic background of the organisms, types of VAGs and antimicrobials, chromosomal mutations, sample type, and colocation of both virulence and resistance genes on single MGEs (Boerlin et al., 2005; Liu et al., 2017; Neamati et al., 2020; Pan et al., 2020; Rezatofighi et al., 2021). In this study, we observed that resistance and virulence genes were randomly distributed among our isolates, suggesting various *E. coli* STs, each with different profiles of resistance and virulence genes, have the potential to cause BSIs. Comparison of ARGs and virulence factors of ST131 and non- ST131 exhibited increased frequencies of six ARGs (*bla*_CTX-M_, *bla*_CTX-M-14_, *bla*_CTX-M-15_, *bla*_OXA_, *bla*_OXA-48_, and *bla*_VIM_) and ten virulence genes (*iutA*, *papG* II, *cnf-1*, *hlyC*, *kpsMT* II, PAI I_536_, PAI II_536_, PAI ICFT_073_, PAI IICFT_073_, and PAI II_J96_) in ST131 (*p* < 0.05). However, *cvaC* and *bla*_OXA-181_ were significantly higher in ST69 (*p* < 0.05). Our results are in line with previous studies that reported a higher prevalence of certain genes in ST69 and ST131 strains (Hojabri et al., 2019b; Hung et al., 2019). Molecular typing is a valuable tool in clinical epidemiology for determining the identical or closely related strains, rule out or confirm clonal outbreaks and sources of infection (Sabat et al., 2013). In our study, REP-PCR differentiated 100 *E. coli* strains into 91 genotypes, suggesting that isolates were clonally unrelated and dissemination of isolates was not due to a clonal outbreak. Additionally, we found that isolates belonging to the same clusters revealed the distinct ARGs and VAGs profiles, suggesting that the horizontal transmission and rearrangement of DNA may have occurred (Aibinu et al., 2012). REP-PCR method also demonstrated that our isolates were genetically diverse and highly heterogeneous. This may be related to genetic variation in our isolates. In a previous study in northeast of Iran, Fallah et al. differentiated the 85 diarrhoeagenic *E. coli* strains into 84 genotypes using REP-PCR (Fallah et al., 2021). Similarly, Ateba and Mbewe found that REP-PCR has more discriminatory power in differentiating *E. coli* strains (Ateba and Mbewe, 2013). However, Foley et al. reported that this method was a limited technique to differentiate among the strains (Foley et al., 2004). In this study, REP-PCR method demonstrated good discriminatory ability in discriminating *E. coli* isolates belonging to same STs. Similar findings have been previously reported (Can et al., 2016; Hojabri et al., 2019a; Lienard et al., 2021; Shahbazi et al., 2023). Although REP-PCR is simple, rapid and cost-effective method, its reproducibility is low and depends on quality of bacterial DNA and the PCR conditions (Foxman et al., 2005). With the purpose of obtaining more reliable and stronger results, sequence-based typing approaches such as multilocus sequence typing (MLST) and whole genome sequencing (WGS) have been developed (Foxman et al., 2005; Vanstokstraeten et al., 2023). MLST is based on the sequencing of conserved housekeeping genes and provides portable, reproducible and an unambiguous data. WGS provides a vast amount of information contained in the genome sequence of bacterial pathogens (Vanstokstraeten et al., 2023); however, the use of WGS in Iran is limited, due to lack of funding and cost, specialized equipment, awareness and education. Moreover, many researchers, physicians and hospitals lack timely access to WGS, constrained by a shortage of specialists as well as clinical workflow and operational challenges. Consequently, conventional methods such as PCR and REP-PCR, along with other cost-effective typing methods, are still used more extensively in Iran. It should be emphasized that the current study has several limitations, including lack of demographics and clinical features of the patients (all isolates studied were from a single hospital in Tehran), antibiotic prescribing data, the small sample size and the lack of other molecular typing methods such as MLST and WGS for further genotypic characterization of these isolates.

## Conclusion

This study demonstrated that high prevalence of MDR septicemic *E. coli* strains with different types of ARGs, VAGs and genotypes is an extremely worrisome sign of BSIs treatment, increases the risk of therapy failure and consequently poses a major threat for hospitalized patients. Active surveillance, stringent prescribing policies, increasing the awareness of ARGs among clinicians and re-defining the infection control measures are essential to curb the dissemination of these strains.

## Data availability statement

The original contributions presented in the study are included in the article/supplementary material, further inquiries can be directed to the corresponding author.

## Ethics statement

The studies involving humans were approved by Ethics Committee of Tehran University of Medical Sciences (ethical approval reference number IR.TUMS.MEDICINE.REC.1401.412). The studies were conducted in accordance with the local legislation and institutional requirements. Written informed consent for participation in this study was provided by the participants' legal guardians/next of kin.

## Author contributions

SaH: Investigation, Methodology, Writing – original draft. ShH: Formal analysis, Software, Writing – review & editing. FJ: Resources, Writing – review & editing. ME: Writing – review & editing. RB: Supervision, Writing – original draft.
